# Case Report: Unusual Peritoneopericardial Diaphragmatic Hernia in an 8-Month-Old German Shepherd Dog, Associated With a Pericardial Pseudocyst and Coexisting Severe Pericardial Effusion Resulting in Right-Sided Heart Failure

**DOI:** 10.3389/fvets.2021.673543

**Published:** 2021-06-07

**Authors:** Imke Hennink, Pia Düver, Ulrich Rytz, Felix Meneses, Melania Moioli, Katja-Nicole Adamik, Alan Kovačević

**Affiliations:** ^1^Department of Veterinary Clinical Medicine, Division of Small Animal Emergency and Critical Care, Vetsuisse Faculty, University of Bern, Bern, Switzerland; ^2^Department of Veterinary Clinical Medicine, Division of Small Animal Surgery, Vetsuisse Faculty, University of Bern, Bern, Switzerland; ^3^Department of Veterinary Clinical Medicine, Division of Small Animal Radiology, Vetsuisse Faculty, University of Bern, Bern, Switzerland; ^4^Department of Veterinary Clinical Medicine, Division of Small Animal Cardiology, Vetsuisse Faculty, University of Bern, Bern, Switzerland

**Keywords:** pseudocyst, case report, German shepherd dog, pericardial effusion, PPDH

## Abstract

**Objective:** This study aims to describe an unusual peritoneopericardial diaphragmatic hernia (PPDH) in an 8-month-old German shepherd dog, associated with a pericardial pseudocyst and coexisting severe pericardial effusion resulting in right-sided heart failure.

**Case Summary:** An 8-month-old, male, intact, German shepherd dog, was referred for ascites. Echocardiography revealed a severe pericardial effusion with a cyst-like structure within the pericardium and consequently decompensated right-sided heart failure. The ascites was secondary to right-sided heart failure (cardiac tamponade). Computed tomography (CT) of the thorax and abdomen was performed and showed PPDH with severe pericardial effusion and presence of a pericardial cyst-like structure; xyphoid cleft and Y-shaped seventh sternebra; and mild thickening along the cranioventral abdominal wall consistent with scar tissue from the previous umbilical hernia surgical repair. During surgery, the PPDH was corrected, and it was revealed that the remnant of the umbilical cord passed through it, into the pericardium. The cyst-like structure was successfully resected and sent for pathology. Histopathology showed signs of a chronic suppurative inflammation, with absence of a mesothelial or endothelial wall layer, thus consistent with a pseudocyst. Based on tomographic and surgical findings, it is suspected that the pseudocyst, together with the pericardial effusion, evolved by an inflammation of the remnant of the umbilical cord during the umbilical hernia surgical repair 1 month prior to presentation. The underlying PPDH most likely favored the development of the pericardial pseudocyst. However, due to prior antibiotic therapy initiated by the private vet, an infectious origin cannot be ruled out completely.

**New or Unique Information Provided:** There are a few case reports describing PPDH and/or pericardial pseudocysts in veterinary patients, but the current case report is unique, since it describes PPDH associated with a pericardial pseudocyst and coexisting severe pericardial effusion resulting in cardiac tamponade. As far as the authors know, such a case has not been described in veterinary medicine before.

## Introduction

Peritoneopericardial diaphragmatic hernia (PPDH) is a congenital anomaly observed in dogs and cats and appears to be a common incidental finding. Its origin lies in a dysembryogenesis resulting in a communicating connection between the pericardial and peritoneal cavity, allowing displacement of abdominal organs into the pericardium. There exist PPDH with a large defect, where parts or whole organs displace into the thoracic cavity, or PPDH with a small diaphragm defect. In small-defect PPDH, only an intra-abdominal fatty pedicle or remnant could enter the pericardial sac and may form a pericardial pseudocyst (1–3). However, the etiopathogenesis of pericardial pseudocysts in animals is still unclear.

Animals with PPDH often have other concurrent congenital abnormalities such as midline defects (e.g., umbilical hernia, cleft palate, sternal abnormalities) and cardiac defects (e.g., ventricular septal defects, subaortic stenosis, pulmonic stenosis, atrial septal defect) (4–8). Peritoneopericardial diaphragmatic hernia is commonly seen as a congenital abnormality in several animals of the same litter (6–9). Therefore, breeding of affected animals is not recommended, and screening of littermates for PPDH should be performed.

One canine case report described a young dog with a cystic lesion-associated congenital PPDH (4). In this 4-month-old golden retriever, one large (17 × 8 × 7 cm) multilobulated cyst-like lesion adherent to the pericardium and the cranial abdominal wall, communicated through the congenital PPDH. The cyst-like lesion was compatible with a pericardial pseudocyst in the early phase of formation, caused by tissue strangulation through the PPDH. Two other case reports describe pericardial pseudocysts as organizing hematomas posttrauma (2, 3). One dog did concurrently also suffer from a PPDH, and it has been assumed that the cystic hematoma arose from trauma to the pericardium-displaced congenital omentum (3).

There are a few case reports describing PPDH and/or pericardial pseudocysts in veterinary patients, but the current case report is unique, since it describes PPDH associated with a pericardial pseudocyst and coexisting severe pericardial effusion resulting in cardiac tamponade. As far as the authors know, such a case has not been described in veterinary medicine before.

## Case Presentation

An 8-month-old, male, intact, German shepherd dog, weighing 32.5 kg, and body condition score of 4/9, was referred for further work-up of ascites. Three days earlier, the dog showed clinical signs of lethargy and anorexia, for which the owners went to the referring veterinarian. Besides a rectal temperature of 39.6°C (103.28°F), the clinical examination at the referring vet was unremarkable. The private vet suspected an infection as underlying cause for the clinical signs and initiated a therapy with meloxicam (0.1 mg/kg q24 h PO; Metacam®, Boehringer Ingelheim GmbH, Basel, Switzerland) and amoxicillin-clavulanic acid (12.5 mg/kg q12 h PO; Clavubactin®, Graeub AG, Bern, Switzerland). In the medical history, the owners mentioned that the dog underwent an umbilical hernia surgical repair 1 month prior to presentation.

During the initial presentation at the authors' institution, the dogs' general condition was good, and he was bright, alert, and responsive. Clinical examination of the dog revealed panting, a heart rate of 160 bpm, muffled heart sounds, moderate femoral pulse quality, rectal temperature of 38.4°C (101.12°F), pink mucous membranes, capillary refill time of 1.5 s, and a distended, non-painful abdomen with a palpable fluid wave. Thoracic and abdominal point-of-care ultrasound (POCUS) showed a severe pericardial effusion with a structure within the pericardial space and a severe peritoneal effusion. A diagnostic sample of the ascites was taken, and cytological examination revealed a pure transudate. On venous blood gas analysis, a mild hyponatremia and hypochloremia were found and refractometric total protein and albumin were mildly decreased (Table 1).

**Table 1:** Blood values at day 1 (initial presentation), day 3 (day of CT and surgery), and day 6 (day of discharge).

**Parameter**	**Day 1**	**Day 3**	**Day 6**	**RI**
Ht (L/L)	0.49	0.43	0.41	0.39–0.57 L/L
Hb total (g/L)	167	141	134	138–204 g/L
Erythrocytes (×10*e*9/L)	NA	6.78	6.47	5.7–9.1 × 10e9/L
MCV (fl)	NA	63	63.7	62.7–74.6 fl
MCH (pg)	NA	20.8	20.7	20.5–24.8 pg
MCHC (g/L)	NA	330	325	316–344 g/L
RDW (%)	NA	13.5	14.2	12.0–13.2%
Reticulocytes (%)	NA	0.87	1.49	0.14–1.48%
Reticulocytes (×10*e*9/L)	NA	59.0	96.5	10.9–111 × 10e9/L
CHr (pg)	NA	22.8	24.9	22.3–27.9 pg
Thrombocytes	NA	439	438	150–400 × 10e9/L
MPV (fl)	NA	13.9	12.2	8.6–14.4 fl
Leukocytes (×10*e*9/L)	NA	16.42	15.09	6–12 × 10e9/L
Normoblasts (×10*e*9/L)	NA	0.00	0.00	0 × 10e9/L
Rods (×10*e*9/L)	NA	0.08	0.30	0–0.3 × 10e9/L
Neutrophils (×10*e*9/L)	NA	12.23	10.11	3–11.5 × 10e9/L
Lymphocytes (×10*e*9/L)	NA	2.22	1.36	1.0–4.8 × 10e9/L
Monocytes (×10*e*9/L)	NA	1.89	2.49	0.15–1.35 × 10e9/L
Eosinophils (×10*e*9/L)	NA	0.00	0.83	0.1–1.25 × 10e9/L
Basophils (×10*e*9/L)	NA	0.00	0.00	0–0.04 × 10e9/L
Sodium (mmol/L)	136.3	140	147	142–154 mmol/L
Potassium (mmol/L)	4.18	4.33	4.48	3.95–5.4 mmol/L
Chloride (mmol/L)	103	101	111	106–118 mmol/L
Calcium ionized (mmol/L)	1.28	NA	NA	NA
Calcium total (mmol/L)	NA	2.57	NA	2.42–2.85 mmol/L
Phosphorus (mmol/L)	NA	1.94	NA	0.91–1.90 mmol/L
Glucose (mmol/L)	5.2	5.37	5.37	4.16–6.69 mmol/L
Cholesterin (mmol/L)	NA	3.82	NA	3.47–10.03 mmol/L
Total protein (g/L)	NA	51.5	NA	55–73 g/L
Refractometric total protein (g/L)	54	NA	NA	60–75 g/L
Albumin (g/L)	26	26.6	27.7	30–41 g/L
Globuline (g/L)	NA	24.9	NA	19–39 g/L
Urea (mmol/L)	NA	4.5	NA	3.3–10.8 mmol/L
Creatinine (μmol/L)	NA	70	74	52–117 μmol/L
Billirubin (μmol/L)	NA	1.9	1.2	0.5–3.9 μmol/L
ALAT (GPT) (U/L)	NA	38	NA	26–126 U/L
AP (U/L)	NA	37	NA	9–132 U/L
ASAT (GOT) (U/L)	NA	17	NA	22–76 U/L
CK (U/L)	NA	73	NA	64–400 U/L
gGT (U/L)	NA	1	NA	<7 U/L
GLDH (U/L)	NA	1	NA	2–10 U/L
Lipase (DGGR) (U/L)	NA	27	NA	24–108 U/L
Canine CRP (mg/L)	NA	103.5	29.9	<10.7 mg/L

*NA, not available; RI, reference interval*.

### Echocardiographic Findings

**Figure 1:**
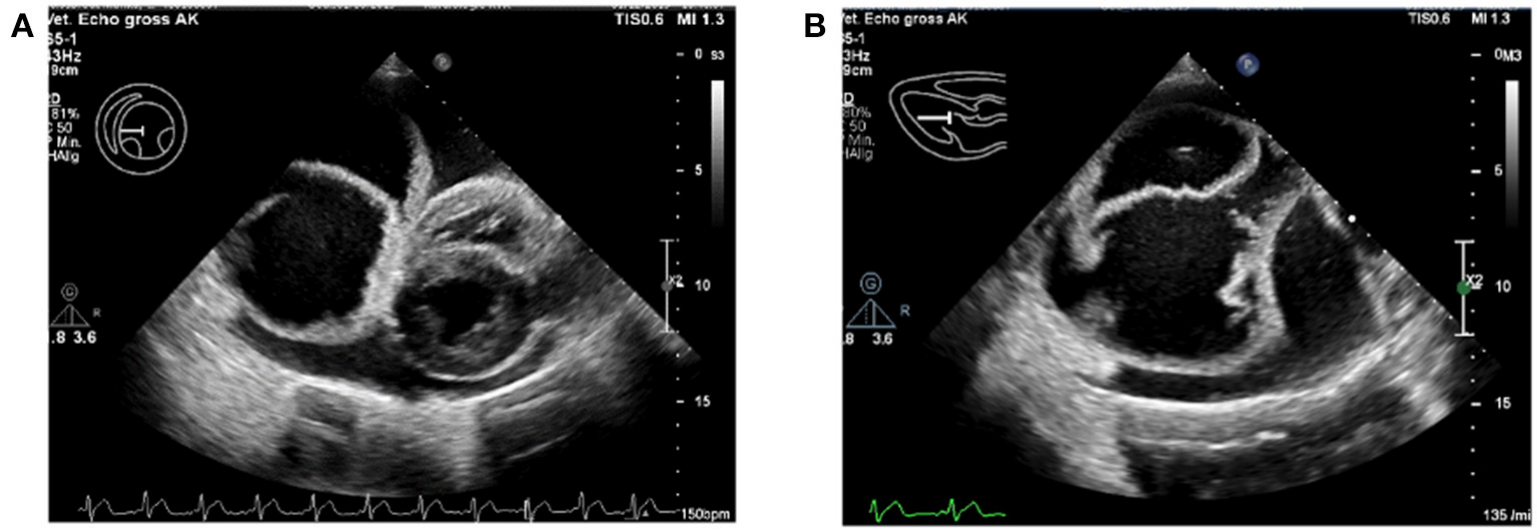
Severe pericardial effusion with the pseudocyst within the pericardium. **(A)** The intrapericardial pseudocyst is seen to the left of the heart. **(B)** Close-up of the intrapericardial pseudocyst, with multiple septate.

As further work-up, an electrocardiogram (ECG) and echocardiography were performed by a board-certified cardiologist (AK). The ECG showed a normal sinus rhythm. On echocardiography, a severe pericardial effusion with an oval-shaped, septate, cyst-like lesion within the pericardial space was found (Figure 1). Since the heart showed signs of decompensation in the form of decreased filling and collapsed right atrium, an emergency pericardiocentesis was performed and a total of 550 ml sanguineous fluid was removed. There were no complications during the procedure, and the hemodynamic parameters normalized, like no collapse of the atria, decrease of the heart rate, and good femoral pulse quality. On an immediate follow-up echocardiographic examination, it was seen that the pericardial effusion was almost completely drained, and the cyst-like lesion remained intact, in both size and form, despite the pericardiocentesis. The pericardial fluid was serohemorrhagic from appearance, with a hematocrit of about 8%. No further diagnostics on the fluid was performed.

At that moment, differential diagnoses for this cyst-like pericardial structure included an idiopathic pericardial (pseudo-)cyst, a congenital pericardial malformation (e.g., PPDH, congenital pericardial cyst), and an *Echinococcosis* (10) infection. The cyst-like lesion was not punctured, because there was a suspicion of, although less likely, an *Echinococcosis* cyst, to avoid spreading of the *Echinococcosis* infection throughout the body.

Based on the stable general condition of the dog, there was a suspicion of a chronic, slowly progressing pericardial effusion, so the dog was not hospitalized and came back for further diagnostics 2 days later, including thoracic and abdominal computed tomography (CT). Depending on the CT findings, surgical removal of the pericardial cyst-like lesion would be scheduled. Deworming with praziquantel (Drontal®, Bayer AG, Leverkusen, Germany) for two consecutive days was initiated, even though an *Echinococcosis* infection was less likely.

### Second Clinical Presentation

During the second presentation at the authors' institution 2 days later, the owner mentioned that in the past 2 days, the dog had been hyporexic and lethargic, developed yellowish diarrhea, and showed exercise intolerance.

On physical examination, the dog showed a respiration rate of 60/min, a heart rate of 120 bpm, muffled heart sounds, moderate femoral pulse quality, rectal temperature of 38.5°C (101.3F°), pink mucous membranes, capillary refill time of 1.5 s, and a distended abdomen with a palpable fluid wave.

An echocardiographic follow-up examination was performed, where recurrence of the pericardial effusion was found, and the cyst-like lesion was unchanged. Cardiac tamponade was present and therefore a second pericardiocentesis was performed. A total of 1,050 ml sanguineous fluid was drained from the pericardial space. The cyst-like lesion, again, remained intact, both in size and shape. The amount of ascites had not been significantly reduced.

### Bloodwork

On complete blood count, mild thrombocytosis, leukocytosis, segmental neutrophilia, monocytosis, and eosinopenia were found. On plasma biochemical analysis, mild decreased sodium and chloride, mild hypoproteinemia and hypoalbuminemia, and increased CRP were present (Table 1).

### Thoracic and Abdominal Computed Tomography

After pericardiocentesis, to stabilize the patient for general anesthesia, a CT of the thorax and abdomen was performed. The CT revealed a Y-shaped seventh sternebra, xyphoid cleft, ventral diaphragmatic defect, and communicating peritoneal and pericardial spaces with a small amount of cranially herniated omentum. There was a marked pericardial effusion with a large thin-walled septate cyst-like structure located in the caudal pericardium displacing the heart cranially and the thoracic caudal vena cava dorsally. Distention of the caudal vena cava and hepatic veins, marked hepatomegaly, and severe ascites were present in the abdomen, and all were consistent with signs of decompensated right heart failure. The dorsal displacement and compression of the thoracic caudal vena cava, by the thoracic cystic lesion, may also have contributed to the congestion of the hepatic veins and the ascites. The cranioventral abdominal wall showed mild focal thickening consistent with scar tissue from the previous umbilical hernia surgical repair. The cyst walls showed mild contrast enhancement, while attenuation of cyst content, pericardial and abdominal effusion was almost isodense to water. A peritoneopericardial hernia and pericardial cyst were diagnosed, and considering the previously repaired umbilical hernia, a more complex midline malformation syndrome was suspected. Based on the diagnosis of PPDH, with the pericardial cyst-like structure being part of it, and the risk for recurrence of right-sided cardiac decompensation without treatment, surgery was performed the same day. The dog did not experience any complications during anesthesia.

### Surgical Procedure

**Figure 2:**
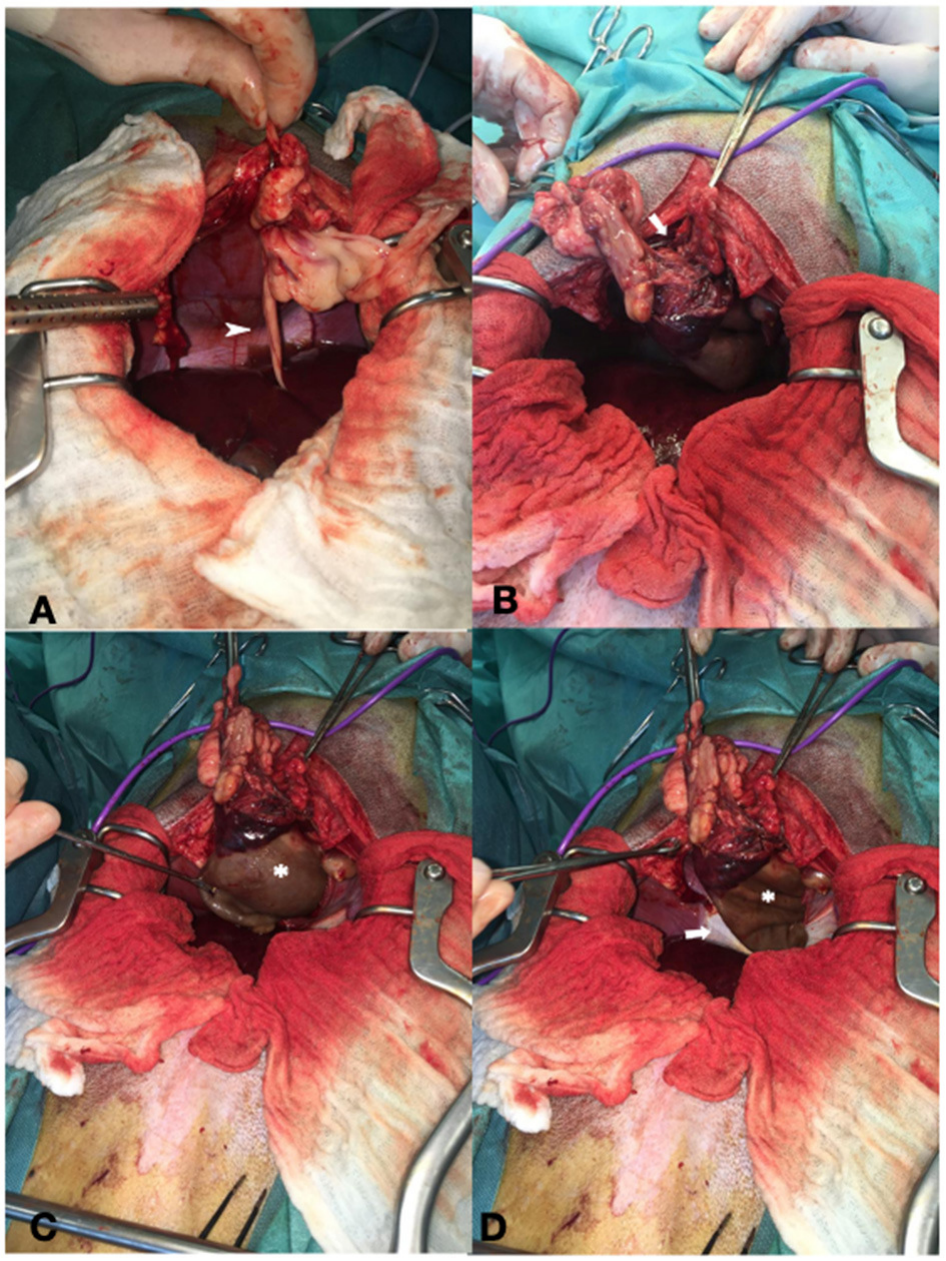
Showing intraoperative findings. **(A)** Fatty ligament containing the umbilical vein remnant (arrowhead). **(B)** Defect of the diaphragm (arrow). **(C)** Cystic lesion cranial to the diaphragm (asterisk). **(D)** Both the diaphragm (arrow) and cystic lesion (asterisk).

A midline celiotomy was performed, and a ventral diaphragmatic malformation was found, through which a fatty ligament, which was probably the ruminate of the umbilical vein, passed into the thoracic cavity. The ligamentous structure was followed, and the small opening in the diaphragm was enlarged, to enter the thoracic cavity. The cyst-like, fluid-filled structure was identified cranially to the diaphragm outside the pericardium even though it was difficult to distinguish between pericardium and cyst-like lesion due to adhesions (Figure 2). The epicardium and pericardium seemed to be roughened. Before removing the structure, it was emptied *via* puncture and a total of 400 ml viscosanguineous fluid was aspirated. The cyst-like structure was removed using a vessel sealing device (LigaSure™, Medtronic, MN, USA). The pericardium was filleted to prevent recurrence of fluid accumulation in the pericardium, causing signs of decompensated right-sided heart failure. Upon closure, a unilateral 5 Fr thoracic drain was placed to enable postsurgical air and fluid removal if needed. The patient did not experience any complications during the anesthesia and was perioperatively treated with ampicillin-sulbactam. No bacteriologic examination of the pericardial fluid or cystic content was performed.

### Hospitalization Period Postsurgery

During hospitalization, the dog was treated with intravenous maintenance fluids (2 ml/kg/h; Plasmalyte®, Baxter, IL, USA), methadone (0.2 mg/kg q4 h IV; Methadon Streuli®, Streuli Pharma AG, Uznach, Switzerland) until 1-day postsurgery and then switched to buprenorphine (15 μg/kg q8 h IV; Bupaq®, Streuli Pharma AG, Uznach, Switzerland) and carprofen (4 mg/kg q24 h IV; Norocarp®, Ufamed AG, Sursee, Switzerland). A probiotic containing *Enterococcus faecium* was added to the therapy, because the dog continued to have mild diarrhea (1 sachet q12 h PO; Purina Veterinary Diets® FortiFlora®, Biokema SA, Crissier, Switzerland). Antimicrobial therapy, initiated by the private vet, was discontinued postoperatively, because we assumed that the origin of the inflammatory response was eliminated by the surgical procedure. The dog's postoperative body weight, without the ascites, decreased by ~2 kg and was 29.8 kg. The patient recovered quickly and started eating again. Since the thorax drain was not productive anymore (no production of fluid or air) and the breathing pattern and effort were both good, the thorax drain was removed on day 2 postsurgery. The following day, the day of hospital discharge, a follow-up echocardiographic examination was performed. No pericardial effusion or other cardiac abnormalities were found, and peritoneal effusion had not recurred. Blood examination still revealed mild leukocytosis, thrombocytosis and monocytosis, mild hypoalbuminemia, and a mildly increased CRP (Table 1). The dog was discharged with carprofen (4 mg/kg q24 h PO; Rimadyl®, Zoetis Schweiz GmbH, Delémont, Switzerland) for two consecutive days.

### Pathology Results

Pathological and histological findings revealed a cystic mass with a size of 11 cm × 6 cm, which was composed of poorly orientated brownish-beige tissue and necrotized material. Histopathological findings revealed that the mass consisted of a chronic, encapsulated process surrounding partly bloody, partly necrotic material with suppurative inflammation and clearly formed granulation. A mesothelial or endothelial wall layer could not be identified, thus classifying the cystic mass to a pseudocyst. No evidence of neoplasia or a bacterial infection was found.

### Follow-Up

A follow-up echocardiographic examination 2.5 weeks and 7 months after hospital discharge were conducted. At both echocardiographic examinations, no cardiac abnormalities were detected and neither the pericardial effusion nor the pseudocyst recurred. The dog was otherwise clinically stable and in a general good condition.

## Discussion

According to the surgical and histopathological findings, there was no evidence of an infectious origin that formed the pseudocyst. However, the pseudocyst might have been formed and/or inflamed during or after the umbilical defect surgical repair. A spreading inflammation *via* the umbilical remnant might have been favored by the presence of the congenital PPDH, and a chronic inflammation may have caused the severe pericardial effusion. The chronicity, hence slow progression of the pericardial effusion, could explain why the dog's general condition seemed good on initial presentation, despite severe pericardial effusion and consequently decompensated right-sided heart failure. The lack of bacterial infection on histopathology can be explained by the antibacterial therapy, initiated by the referring veterinarian. So, an infectious origin cannot be completely ruled out.

In veterinary literature, there exists one other canine case report describing a young dog with a pericardial pseudocyst in association with congenital PPDH, although pericardial effusion and consequent decompensated right-sided heart failure was not present (4). In this 4-month-old golden retriever, the right medial and quadrate lobes of the liver together with one large (17 × 8 × 7 cm) multilobulated cyst-like lesion, adherent to the pericardium and the cranial abdominal wall, communicated through the congenital PPDH. Histopathology of the cystic lesion revealed a layer of fibrous stroma and granulation tissue, with a small amount of fibrin present in the lumen, compatible with a pericardial pseudocyst in the early phase of formation, caused by tissue strangulation through the PPDH.

Pericardial pseudocysts are described in canine and feline patients (1–4, 11). However, the etiopathogenesis of pericardial pseudocysts in animals remains unclear. Similarly, as in our case report, these lesions are almost always connected to an intra-abdominal fatty pedicle or remnant, which could have entered the pericardial sac through a small PPDH and are often connected to the cranial part of the pericardium (1–3). There are two canine case reports describing quite similar clinical presentations of a dog with a pericardial pseudocyst. The first case report describes a mixed-breed dog (4 years old) with severe cardiac tamponade secondary to a pericardial pseudocyst, although this dog did not have a congenital PPDH. The pericardial pseudocyst was consistent with a chronic cystic hematoma, and it was likely formed due to previous trauma (2). The second case report describes a miniature Schnauzer (2 years old), 6 months postvehicular trauma, presented with muffled heart sounds on the right side and enlarged cardiac silhouette on thoracic radiographs. This dog showed a small congenital PPDH, with herniated mesenteric fat passing through it, into the pericardial sac and adhered to a well-circumscribed, multilobular mass (8 × 6 × 5 cm). The pseudocyst was consistent with an organizing hematoma posttrauma (3). Although the relationship between the bleeding episode that created the hematoma and the congenital PPDH was not clear, the authors have assumed that the cystic hematoma arose from trauma to the pericardium-displaced congenital omentum.

True pericardial cysts in dogs are very rare and only two veterinary case reports describe true pericardial cysts in dogs (1, 12). In humans, four different true congenital pericardial cysts are described: coelomic, lymphangiomatous, bronchial, and teratomatous. The wall of these true cysts contains a layer of endothelium or mesothelium (1). The cyst-like lesion reported in the current case report did not contain an endothelial or mesothelial wall, thus is consistent with a pericardial pseudocyst.

Besides pericardial (pseudo-)cysts, there exist, although extremely rare, congenital cardiac cysts (13, 14). In humans, cardiac cysts contain, like true pericardial cysts, a wall with a mesothelial or endothelial layer. In the veterinary literature, there is one case report that describes two West Highland white terriers (16 weeks old) with multiple congenital cardiac cysts in the left ventricular wall and some connected with the ventricular lumen. Both dogs were euthanized due to severe decompensated heart failure. Postmortem findings of both dogs revealed that the wall of these cysts contained an endothelial layer, consistent with true cardiac cysts. However, the etiopathogenesis of these congenital cardiac cysts is still unknown and also hard to establish since the disease is extremely rare.

Based on tomographical and surgical findings, the described physical abnormalities do have some similarities with incomplete pentalogy of Cantrell. The description of complete pentalogy of Cantrell in humans consist of a defect (cleft) of the inferior sternum, a midline supra-umbilical abdominal wall defect, a congenital intracardiac anomaly, a deficit of the anterior diaphragm, and a defect in the diaphragmatic pericardium. There are three classifications of the pentalogy of Cantrell. In class 1, patients have all five defects, patients with class 2 have four defects, including intracardiac and ventral wall anomalies, and patients with class 3, also known as the incomplete expression, have various combinations of abnormalities including a sternal defect (4, 5). To date, in veterinary medicine, this classification does not exist. In the case report described here, only four of the total five defects were present, namely, xyphoid cleft and Y-shaped seventh sternebra, midline supra-umbilical abdominal wall defect, defect of the anterior diaphragm, and defect of the diaphragmatic pericardium, and this would allow the classification into class 3, incomplete, pentalogy of Cantrell.

In veterinary literature, to date, only three veterinary case reports exist that describe incomplete pentalogy of Cantrell. Two case reports involve a young German shepherd dog (15, 16), and one case report involves a 1-year-old cat (17). All these three cases involved young animals, affected with PPDH, but none have reported severe pericardial effusion with a pericardial cyst-like structure. It is interesting that the two other canine case reports about pentalogy of Cantrell, present in the literature, are describing this anomaly in the German shepherd dog as well (15, 16), suggesting that it is likely an abnormality occurring more often in this breed. In humans, it is suspected to have a multifactorial inheritance (18). In the German shepherd dog, pentalogy of Cantrell may also have a hereditary background.

## Conclusion and Clinical Relevance

This case report describes an unusual PPDH associated with a pericardial pseudocyst and coexisting severe pericardial effusion resulting in cardiac tamponade. All dogs with a PPDH could possibly develop an intrapericardial pseudocyst, originating from an intra-abdominal fatty pedicle or remnant, which could have entered the pericardial sac through a small PPDH. Prior abdominal surgery in an undetected PPDH might favor ascending inflammation by a communicating fatty pedicle or remnant through the PPDH and consequently form a pseudocyst or, as in this case, even severe pericardial effusion. Clinical signs differ and depend on the severity of the PPDH (number and size of organs displaced intrapericardially), the chronicity, size of the intrapericardial pseudocyst, and the presence and severity of pericardial effusion and consequently cardiac tamponade. In all young animals, with history of prior abdominal surgery (e.g., umbilical hernia surgery), presenting with signs of right-sided heart failure, PPDH, and the presence of pericardial pseudocysts with/without pericardial effusion, should be considered.

## Data Availability Statement

The original contributions generated for the study are included in the article/supplementary material, further inquiries can be directed to the corresponding author/s.

## Author Contributions

IH performed emergency care of the patient and wrote the paper. AK performed the echocardiographic examinations on admission and on follow-up appointments, he also contributed to the final version of the manuscript. PD performed the surgery and contributed to the final version of the manuscript. K-NA supervised IH during the emergency care of the patient. UR performed the surgery together with PD. FM performed analysis of the CT images of the patient. MM performed analysis of the CT images of the patient and contributed to the final version of the manuscript. All authors contributed to the article and approved the submitted version.

## Conflict of Interest

The authors declare that the research was conducted in the absence of any commercial or financial relationships that could be construed as a potential conflict of interest.
